# Commentary: Techniques and graft materials for repairing peripheral nerve defects

**DOI:** 10.3389/fneur.2024.1420324

**Published:** 2024-06-21

**Authors:** Elena Stocco, Silvia Barbon, Aron Emmi, Cesare Tiengo, Raffaele De Caro, Veronica Macchi, Andrea Porzionato

**Affiliations:** ^1^Section of Human Anatomy, Department of Neuroscience, University of Padova, Padova, Italy; ^2^Department of Women's and Children's Health, University of Padova, Padova, Italy; ^3^Department of Surgery, Oncology and Gastroenterology, University of Padova, Padova, Italy; ^4^Foundation for Biology and Regenerative Medicine, Tissue Engineering and Signaling, Onlus, Padova, Italy; ^5^Plastic Surgery Unit, Department of Neuroscience, University of Padova, Padova, Italy

**Keywords:** peripheral nerve injury, nerve conduits, luminal filler, bioactivation, tissue engineering

## 1 Introduction

We read with interest the review article by Zou et al. (1) entitled “*Techniques and graft materials for repairing peripheral nerve defects”*. Here, the Authors discuss current approaches to peripheral nerve injury (PNI) with substance loss (autograft/allograft/nerve conduit), also highlighting methods' advantages and disadvantages, following our previous contribution (2). In addition, appealing materials for nerve conduits (NC) fabrication were overviewed too (1), highlighting that their intrinsic characteristics can affect the regeneration outcome (1, 2).

Certainly, while looking for the ideal device, we believe that together with a focus on conduit material, conduit topography must be considered too, as significantly modulating nerve regeneration across the gap. Based on that, design strategies providing nerve guides with specific topographic cues will be described and discussed below.

## 2 Nerve conduits designs

### 2.1 Nerve conduits with porous wall

According to size, biomaterials pores can be classified as macropores (100–500 μm), micropores (< 100 μm), and nanopores (< 1,000 nm). Surely, wall pore dimension affects the device inner environment; acting as a strain, a porous wall can select the molecules that can be exchanged between the regenerated peripheral nerve and the surrounding environment, thus influencing cell viability/proliferation/blood vessels infiltration (3, 4).

Fully permeable (pore diameter: >50 μm), semi-permeable (pore diameter: < 10 μm), and asymmetric (external surface pore size > lumen surface pore size) conduits have been developed to overcome autograft issues in case of severe PNI. These nerve guides showed to improve the neuro-regenerative process under different aspects. Specifically, semi-permeable conduits can avoid fibrous scar formation; fully-permeable conduits can promote direct signal communication between cells; asymmetric structures-based conduits can assure for efficient removal of metabolic waste. Nevertheless, some intrinsic disadvantages related to their use still need to be addressed, including no direct signal communication between cells affecting semi-permeable and asymmetric structures and the risk of fibrous infiltration concerning fully-permeable devices (3).

Porosity and pore size are often dependent on scaffold fabrication method (4); conduits fabricated using traditional methods are mainly characterized by pores with random morphology. Hence, lithography processes, electrospinning, and 3D printing technology are gaining researchers' attention, distinguishing as promising techniques in exerting control over wall porosity dimension/organization (2).

### 2.2 Nerve conduits with grooved wall

Schwann cells (SCs) growth direction and migration in the postinjury microenvironment exert a fundamental role in promoting axon growth. Thus, the development of conduits displaying a regular topology can discourage random distribution of SCs (5). NC eventually endowed by anisotropic topographical cues, consisting in micro- and nanogroove patterns in the luminal wall, have received extensive attention. In fact, patterns can trigger a contact guidance effect, thus instructing cells to align along a preferential direction and significantly influencing neurite outgrowth, cellular alignment, elongation and differentiation (6–8).

Thin microgrooves (5–10 μm) confine the growth of cells and axon elongation; grooves with widths and spacings of 10–20 μm favorably support SC adhesion and proliferation; patterns comparable with cell dimensions (10–50 μm) are appealing too. The narrower microgrooves were found to improve axonal alignment, diminish the number of axon branches per cell, and decrease incorrect distal re-connection (2, 9). Groove depth can also affect surface/cells interaction. To support neurite alignment or outgrowth, depths ranging from 1 to 4 μm are adequate; conversely, sizes inferior to 300 nm revealed to be less favorable. Difficulties in fabrication are the main critical issue in the development of grooved conduits (2, 10). Various approaches have been developed to produce scaffolds with anisotropic structure, including electrospinning, lithography, and 3D bioprinting (7).

### 2.3 Nerve conduits with multichannel

A multichannel conduit may be more likely to preserve the linear organization of regenerating axons in the peripheral nerve (10).

Introduction of macroscale, longitudinally oriented multichannels within NC can guarantee a permissive pathway for axon growth, favoring SCs' attachment, as well as growth factors' release. Contextually, they can reduce the dispersion of regenerating axons avoiding their own consequent misdirection, eventually associated with different target polyinnervation (11, 12). According to the literature, the number/diameter of the channels within a single conduit may vary, ranging from 1 channel/conduit (13) to over 100 (14). Certainly, the presence of multichannels within the conduits provides for a regenerative environment which better mimics the native nerve fascicular structure (2).

Different methods have been adopted for multichannel conduit fabrication, including solvent or thermally induced phase separation, injection molding, electrospinning, and 3D printing (15).

### 2.4 Nerve conduits with fillers

Introducing luminal fillers into conduits aims not only to enhance conduit-associated outcomes at shorter distances but also to improve nerve reconstruction in case of wide gaps (16). Several studies report the incorporation of macro-, micro-, and nanoscale filler materials within the NC to encourage axon regeneration (12). Different natural (e.g., fibrin, collagen, laminin, and agarose) and synthetic polymers [e.g., polyamide, polyacrylonitrile-co-methylacrylate, polyglycolic acid, poly-L-lactic acid, and poly(lactic-co-glycolic acid)] have been used as solutions, hydrogel filaments, porous sponges or films (2, 9, 17, 18).

Recently, attention has been directed to Self-Assembling Peptides (SAPs), mimicking ECM organization. SAPs consist in short peptide molecules self-organizing into stable secondary structures (α-helix, β-sheet, or random coil) and further forming various aggregation states (fibrils, fibril networks, membranes, and gels) under *in vivo* pH and ion concentration (2, 19–25). Additionally, SAPs functionalization with the laminin-derived peptide IKVAV (sequence: isoleucine–lysine–valine–alanine–valine) (19–21, 23), or brain-derived neurotrophic factor (BDNF) (21) have been also used to improve outcomes of chitosan or poly(L-lactide) (PLLA) conduits.

## 3 Discussion

Intense efforts are dedicated toward the fabrication of the ideal NC. Certainly, the biomaterial adopted for devices' fabrication has a fundamental role over their outcomes; interestingly, a blend of synthetic and natural polymers can assure for appropriate structural/mechanical support (synthetic polymer) and biomimicry (natural polymer) (26). However, providing topographic cues is essential too. Porous and grooved walls, multi-channel conduits, and NC with fillers (in the form of fibers or hydrogels) are among the most appealing strategies for conduits structure/ultrastructure bioactivation (2, 27). The selected biomaterial and the desired topographical cues to be introduce will critically guide toward the fabrication technique to be adopted (12).

## Author contributions

ES: Writing – original draft, Conceptualization. SB: Writing – original draft. AE: Writing – review & editing. CT: Writing – review & editing. RDC: Writing – review & editing, Supervision. VM: Writing – review & editing, Supervision. AP: Writing – review & editing, Conceptualization.
